# Assessing the Dermal Epidermal Junction of Hyperkeratotic Actinic Keratoses via Line‐Field Confocal Optical Coherence Tomography

**DOI:** 10.1111/ijd.70328

**Published:** 2026-02-11

**Authors:** Simone Michelini, Pietro Scribani Rossi, Giuseppe Gemma, Flavia Persechino, Giordano Vespasiani, Claudio Conforti, Francesco Ricci, Carmen Cantisani, Giovanni Pellacani

**Affiliations:** ^1^ Dermatology Clinic, Department of Medical and Cardiovascular Sciences Sapienza University of Rome Rome Italy; ^2^ IRCCS Istituto Dermopatico Dell'immacolata (IDI‐IRCCS), Dermatological Research Hospital Rome Italy; ^3^ Department of Life Science, Health and Health Professions Link University of Rome Rome Italy

**Keywords:** actinic keratosis, line‐field confocal optical coherence tomography, noninvasive diagnostics, PRO‐score

## Abstract

**Introduction:**

Actinic keratoses (AKs) are ultraviolet (UV)‐induced skin lesions that may progress to invasive squamous cell carcinoma. Hyperkeratotic AKs hinder line‐field confocal optical coherence tomography (LC‐OCT) imaging, limiting assessment of the dermal‐epidermal junction (DEJ) and PRO‐score, a histopathological index of dermal invasion.

**Objective:**

The aim of this study was to evaluate whether moisturization improves DEJ visualization and PRO‐score evaluation in hyperkeratotic AKs using LC‐OCT.

**Materials and Methods:**

Fifty‐nine hyperkeratotic AKs were randomized to receive either 20‐min tap water occlusion or 20% salicylic acid ointment (SAO). LC‐OCT scans were taken before and after treatment. DEJ visibility, PRO‐score (0–3), and confidence in PRO assignment (cPRO) were rated by blinded evaluators.

**Results:**

DEJ visibility significantly increased posttreatment (from 39.0% to 86.4%, *p* = 0.015), with no significant difference between water and salicylic acid ointment (SAO). Confidence in PRO‐scoring also improved (*p* < 0.001), and 39.1% of lesions with initial scores were reclassified. G2 AKs had higher DEJ visibility than G3 both before and after treatment.

**Conclusion:**

Moisturization enhances LC‐OCT evaluation of hyperkeratotic AKs, improving DEJ visualization and diagnostic confidence.

## Graphical abstract

Actinic keratoses (AK) are ultraviolet (UV)‐correlated precursor lesions of invasive cutaneous squamous cell carcinoma (iSCC). AK thickness does not correlate with increased iSCC transformation, but dermal‐epidermal junction (DEJ) protrusion does. The DEJ of hyperkeratotic lesions is harder to study via line‐field confocal optical coherence tomography (LC‐OCT). The DEJ of 59 AK was analyzed before and after treatment with water or salicylic acid ointment (SAO) for 20 min. Treatment, irrespective of the solution, increased DEJ visibility and diagnostic confidence.

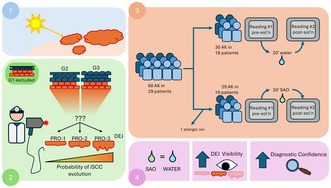

## 1 Introduction

Actinic keratoses (AK) are ultraviolet (UV)‐induced skin lesions that may progress into infiltrative cutaneous squamous cell carcinoma (iSCC) [1, 2, 3, 4]. AK classification may be based on clinical as well as histopathological aspects. Olsen et al. [5] differentiated AKs clinically into three grades, based on the degree of hyperkeratosis measured by thickening and palpability, whether mild (G1), moderate (G2), or severe (G3). Cockerell combined clinical and histological manifestations, identifying KIN‐I as smooth, flat, erythematous macules/patches with lower third involvement of epidermis, KIN‐II pink to erythematous papules/plaques with variable roughening with lower two‐thirds involvement, KIN‐III as red‐scaly plaques, with adnexal and full thickness epidermal involvement [6]. Rowert‐Huber et al. [7] proposed classifying AKs solely on the extent of epidermal involvement, defining them as “early in situ SCC type” AK I/II/III.

While the notion that AK are precursor lesions to iSCC is generally agreed upon, which AK will progress into iSCC is not predictable [8]. Correlation between AK grading and iSCC evolution is debated; while evidence has been presented suggesting greater risk in Olsen G3 compared to G1 and G2, others dispute this notion [9]. One report suggests that an increased AK grade was not correlated with pathology keratinocyte dysplasia or P53 mutation [10]. Another reports that lower Olsen‐grade AKs correlated with a greater degree of dysplasia, suggesting AK do not necessarily, nor often, progress from a low to high grade to become iSCC [11]. The PRO score, another pathology‐based classification system, has been suggested which evaluates atypical keratinocyte protrusion into the dermis [12, 13]. Protrusions are described as crowding (PRO‐I), budding (PRO II), and papillary sprouting (PRO‐III) [12]. No correlation between PRO score and Olsen AK grading was found; yet AKs with higher PRO score (II and III) were most commonly found adjacent to iSCCs [12, 14].

Pathology classifications may increase in relevance in clinical settings due to the advent of various noninvasive instrumental techniques, such as line‐field confocal optical coherence tomography (LC‐OCT) [15, 16]. LC‐OCT simultaneously acquires both clinical and vertical or horizontal scans of the epidermis and has gained a growing role in dermatological disease detection, diagnosis, treatment, and follow‐up after treatment [17, 18]. Ruini et al. [19, 20] reported in a pilot study that LC‐OCT could be used to study the PRO classification in vivo of AK contributing to diagnosis and therapeutic follow. The DEJ of hyperkeratotic AKs was typically not fully evaluable due to the increased refractive index, scales, and air bubbles, limiting the instrumental PRO‐score estimation. Reduced interobserver agreement in LC‐OCT–based DEJ assessment, likely influenced by lesion hyperkeratosis and acanthosis, is not confined to AKs but has been reported across nonmelanoma skin cancer lesions [21].

The aim of this study is to establish whether a rapid moisturization of hyperkeratotic AKs can improve dermal epidermal junction evaluation and PRO‐score classification by means of LC‐OCT.

## 2 Materials and Methods

This study sought to examine 60 AKs in patients from Rome's Policlinico Umberto I; consecutive hyperkeratotic AKs, either G2 or G3, were included in the study.

Each AK was first scanned using a commercially available, hand‐held LC‐OCT scanner (deepLiveTM, DAMAE Medical, Paris, France; 800 nm, axial and lateral resolution < 1.3 μm; field of view 1.2 mm × 0.5 mm × 0.5 mm), then randomized to be treated 20 min with either tap water in occlusion (via a disk of absorbent paper in Finn‐chamber) or 20% salicylic acid ointment (SAO). After 20 min the occlusive apparatus or ointment was gently removed, and a second LC‐OCT reading was taken immediately after. Co‐localization of LC‐OCT scans acquired before and after treatment was ensured by centering the probe on a dermatographic marker placed on each AK lesion, using the device's internal dermatoscope.

Each reading was assessed blindly. Two evaluators were asked to agree on three criteria: visibility of the dermal‐epidermal junction (DEJ), PRO‐score, and PRO‐score diagnostic confidence (cPRO). If an agreement was not reached, a third evaluator was asked to settle the score.

DEJ visibility was classified as either “not visible” or “visible.” Only in cases where DEJ was considered “visible,” PRO score was rated on a scale from 0 to 3, where 0 indicated linearity, 1 represented mild deviation from linearity, 2 indicated moderate undulations, and 3 denoted strong undulations [12]. cPRO was a subjective score ranging from 1 to 3, assessing the evaluator's confidence in the PRO score, rating 1 for uncertainty, 2 for moderate certainty, and 3 for high certainty in the assigned PRO score. Differences between scores before and after treatment were analyzed using SPSS (Statistical Package for Social Science).

A *χ* ^2^‐test of association was performed to confirm that G2 and G3 were equally as likely to be treated with either solution. *X* ^2^‐test was performed to study the difference between DEJ visibility in either solution, both prior to and after treatment. A Wilcoxon signed‐rank test was performed to analyze changes in PRO‐score and cPRO both before and after treatment. Analyses were also performed within G2 and G3 AK subgroups to examine whether AK grade influenced readings. A *p*‐value < 0.05 was considered statistically significant.

## 3 Results

**FIGURE 1:**
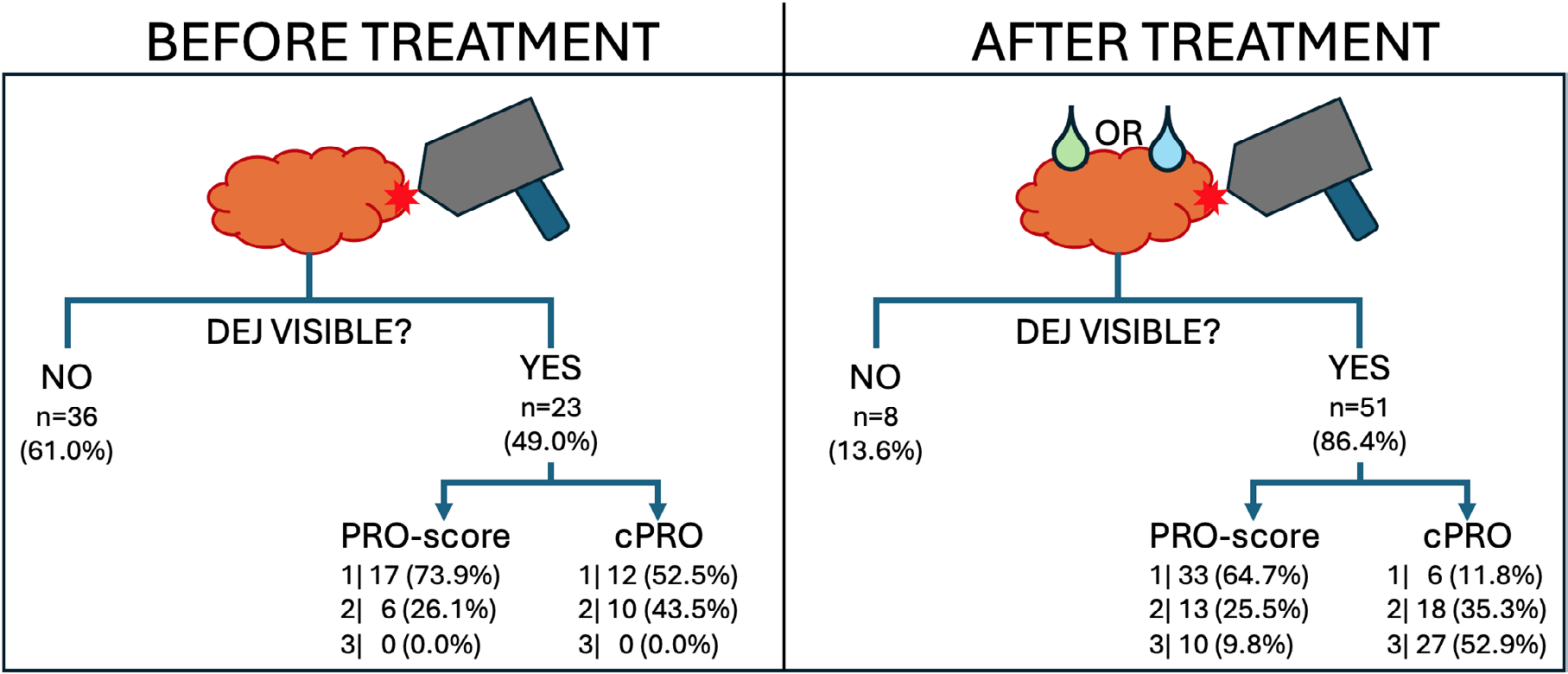
Visual representation in hyperkeratotic AK before and after hydration with water or SAO. Number and percentage of lesions in which the DEJ was visible by LC‐OCT, and the distribution of PRO‐score and cPRO values when the DEJ was identifiable.

Sixty hyperkeratotic AKs were found on a total of 29 patients. All AKs were randomly assigned one of the two moisturizing procedures; 30 were to be treated with water and 30 with SAO. One patient with a single AK in the SAO cohort was excluded due to a local reaction reported during LC‐OCT second acquisition and was subsequently excluded from data collection. Statistical analysis was consequently performed on a total of 59 AKs, 30 treated with water and 29 with SAO (Figure 1).

Of the AKs (*n* = 59), 64.4% (*n* = 38) were Olsen G2, while 35.6% (*n* = 21) were G3. The water assigned cohort had slightly more G3 AK than the SAO group (12 vs. 9); however, this difference was not significant (*p* = 0.361).

At baseline, before treatment, only 39.0% (*n* = 23) of all AKs had a visible DEJ and could receive a PRO score and cPRO score. Of these, 17 (73.9%) had a PRO score of 1, and 6 (26.1%) had a score of 2; none scored 3; over half (*n* = 12) had a low confidence score and only one high certainty. There was no significant difference between the two groups (water vs. SAO) in baseline DEJ visibility (*p* = 0.218), PRO score (*p* = 0.735), or cPRO (*p* = 0.712).

After treatment, DEJ visibility significantly increased to 86.4% (*n* = 51; *p* = 0.015). The solution used did not influence DEJ visibility (*p* = 0.478), PRO score (*p* = 0.239), or cPRO (*p* = 0.202).

Among AKs with a visible DEJ before treatment, 9 out of 23 (39.1%) had a change in PRO score after treatment: 21.7% (*n* = 5) increased and 17.4% (*n* = 4) decreased. Also, cPRO was significantly higher after treatment (*p* < 0.001).

Considering the different Olsen's degrees, G2 AK were more likely to have a visible DEJ compared to G3 both before (19 vs. 4, *p* = 0.020) and after treatment (36 vs. 15, *p* = 0.012). No significant correlation was found between AK grade and PRO score before (*p* = 0.231) or after treatment (*p* = 0.093).

## 4 Discussion

The categorization of AKs as precancerous lesions or in situ cancers is strongly debated [1, 5, 6, 7]. AK has been historically classified either clinically or pathologically based on the extent of atypical keratinocyte invasion of the epidermis from the basal layer. These classifications do not correlate with AK progression into iSCC [8, 9]. The PRO‐score is a pathological scoring system that concentrates on atypical keratinocyte protrusion within the dermis [12]. While PRO‐score is not correlated to the Olsen grade, it seems to have a stronger correlation with transformation into iSCC [12, 14].

Among available noninvasive techniques, LC‐OCT allows clinicians to estimate the AK PRO‐score due to high‐resolution imaging of vertical sections of skin and 3D reconstruction [16]. Nonhypertrophic AK have been compared pathologically and instrumentally via LC‐OCT [5, 19]. Exclusion of hyperkeratotic lesions is likely due to the refractive effect of air and scales, as well as an increased distance from the DEJ, which impairs the visualization of DEJ [20].

The objective of this study was to determine whether moisturization of hyperkeratotic AK eased DEJ evaluation, and consequently PRO‐score and cPRO evaluation. A short contact occlusion with tap water or application of SAO for 20 min was chosen for their different mechanism of moisturization and for easy availability.

**FIGURE 2:**
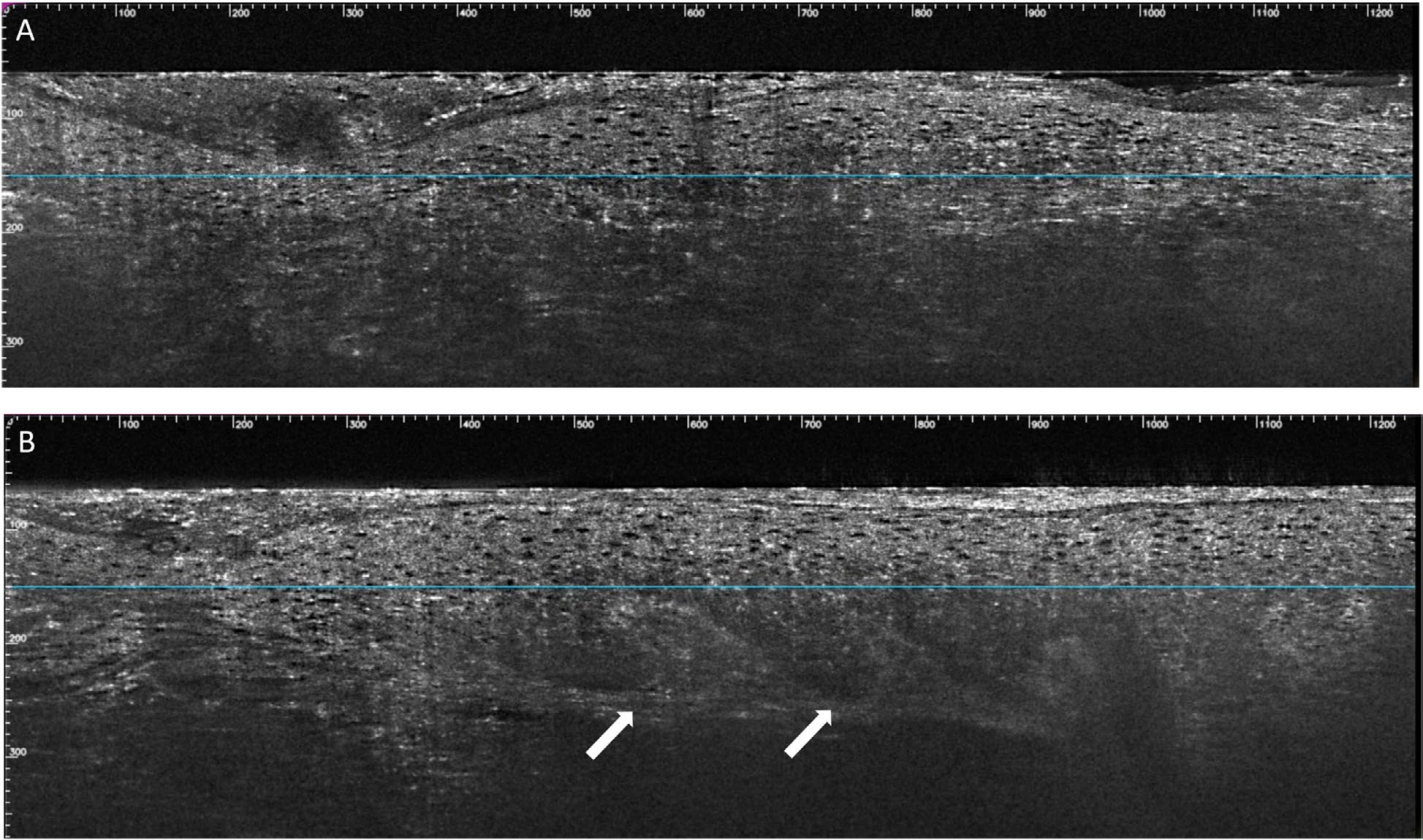
DEJ visibility in G2 AK treated with water. (A) before treatment, DEJ is not clearly visible, (B) after treatment, a PRO‐score of 3 was assigned as DEJ was more readily visible (arrows).

After short‐term application of both moisturizing agents, DEJ was visible in a significantly higher number of cases (from 39% to 86%) (Figure 2). G2 AK responded better to treatment with a 94.7% DEJ visibility after treatment compared to a 71.4% G3 AK response. Probably, the thicker layering of G3 AK impairs effective LC‐OCT readings by increasing reflectance and casting shadows on deeper layers, also after short‐term application of moisturizing agents. In more hyperkeratotic AKs, the DEJ may lie beyond the depth of the LC‐OCT field of view, precluding visualization and assessment.

A significant increase in cPRO was observed after treatment. Nearly 40% of AKs with a baseline PRO score required reclassification after treatment; all of these had an initially low confidence. AKs were nearly equally likely to increase (*n* = 5) or decrease (*n* = 4) in PRO score. This further suggests that moisturizing AKs is a simple action which allows for a more confident PRO scoring. Interestingly, no significant difference was observed between the applied moisturizing agents. The water likely contributed to greater visibility by hydrating the scale, while the SAO exposure may not have been sufficient to carry out a keratinolytic effect, but the ointment's lipophilic nature may have allowed scale adherence and reflectance reduction.

These data suggest that treating AKs with hyperkeratosis prior to LC‐OCT readings allows us to study the DEJ more confidently, better estimating the degree of budding, as well as assigning a PRO score. The type of solution, per se, had no influence on DEJ visibility, PRO score, or the cPRO. Water is typically more readily available, though we found that the application of SAO was simpler in our clinical context, with no need for any occlusive apparatus. However, 14% of AKs remained unreadable, mostly being G3 Olsen AKs, suggesting that in these cases a longer application of moisturizing agents should be recommended.

This study has several limitations, starting with its small sample size. Another limitation is the lack of histopathological confirmation to validate instrumental findings. Also, only a short application of two moisturizing agents was tested.

Further studies comparing SAO to inert ointments or other moisturizing agents may be necessary to investigate this effect. Different approaches can be used to reduce hyperkeratosis, either physical, such as superficial curettage or laser, or chemical, (e.g., lytic agents). However, it should be considered that bleeding, a frequent consequence of superficial curettage, hampers LC‐OCT tissue examination, and strong lytic agents should be handled with precaution. Scan co‐localization was achieved using the probe's internal dermatoscope; a software‐based mapping tool was not employed, as it was not available at the time of data acquisition. Finally, future studies may evaluate whether shorter exposure times are sufficient to improve DEJ visibility or whether one medium may achieve visibility more rapidly.

This study shows that treating AK, especially if G2, prior to LC‐OCT readings is a simple and rapid procedure that increases DEJ visibility and confidence in diagnosis.

## Funding

The authors have nothing to report.

## Conflicts of Interest

The authors declare no conflicts of interest.

## Data Availability

The data that support the findings of this study are available on request from the corresponding author. The data are not publicly available due to privacy or ethical restrictions.
